# The Relationship between Self-Reported Exposure to Sugar-Sweetened Beverage Promotions and Intake: Cross-Sectional Analysis of the 2017 International Food Policy Study

**DOI:** 10.3390/nu11123047

**Published:** 2019-12-13

**Authors:** Hannah Forde, Martin White, Louis Levy, Felix Greaves, David Hammond, Lana Vanderlee, Stephen Sharp, Jean Adams

**Affiliations:** 1 Centre for Diet & Activity Research (CEDAR), MRC Epidemiology Unit, University of Cambridge School of Clinical Medicine, Box 285 Institute of Metabolic Science, Cambridge Biomedical Campus, Cambridge CB2 0QQ, UK; Martin.White@mrc-epid.cam.ac.uk (M.W.); Stephen.Sharp@mrc-epid.cam.ac.uk (S.S.); jma79@medschl.cam.ac.uk (J.A.); 2 Public Health England, Wellington House, 133-155 Waterloo Road, Lambeth, London SE1 8UG, UK; Louis.Levy@phe.gov.uk (L.L.); Felix.Greaves@phe.gov.uk (F.G.); 3 School of Public Health and Health Systems, University of Waterloo, Waterloo, ON N2L 3G1, Canada; david.hammond@uwaterloo.ca (D.H.); lana.vanderlee@uwaterloo.ca (L.V.)

**Keywords:** sugar, sugar-sweetened beverages, soft drinks, marketing, promotion, advertising

## Abstract

Sugar-sweetened beverage (SSB) consumption is independently associated with several non-communicable diseases, so policymakers are increasingly implementing measures, such as marketing regulation, to reduce intake. To help understand how such measures work, this study examined the association between SSB consumption and self-reported exposure to SSB promotions, both overall and by type of promotion, and whether these relationships vary between the UK, USA, Canada, Mexico, and Australia. Cross-sectional analysis of the online 2017 International Food Policy Study was performed (*n* = 15,515). Participants were grouped into 5265 (34%) non-, 5117 (33%) low-, and 5133 (33%) high-SSB consumers. Multinomial logistic regression models examined whether SSB consumption varied by exposure to total SSB promotion and by type: traditional, digital, recreational environment, and functional environment. Multiplicative interactions were included to investigate international variations. An additional unit of total self-reported SSB promotion exposure increased the likelihood of participants being low SSB consumers (relative risk ratio (RRR) = 1.08, 95% confidence interval (CI) = 1.06–1.10) and high SSB consumers (RRR = 1.13, 95% CI = 1.11–1.16). Only exposure to traditional and digital promotion increased the likelihood of participants being SSB consumers, though this may be explained by degree of exposure, which was not measured in this study. Some evidence illustrated international variation in these relationships.

## 1. Introduction

In 2017, 11 million deaths worldwide were attributable to dietary risk factors [1]. High consumption of sugars [2,3] is a known risk factor for non-communicable diseases (NCDs), such as overweight [4] and type II diabetes [5], cardio-metabolic risks [6], poor oral health [7], and overall mortality [8]. Sugar sweetened beverages (SSBs) are a substantial source of dietary sugar and the greatest source of dietary sugar for young people in many countries worldwide [9,10,11,12,13]. Consumption of SSBs is associated with several NCDs, independent of effects mediated by obesity [14,15]. Understanding determinants of SSB consumption could inform interventions to reduce SSB intake and thus prove beneficial to diet-related health.

Many attribute the scale of global SSB consumption at least in part to successful marketing [16,17]. Significant attention has been paid to the effects of product promotion [18]—a firm’s persuasive communication [19]—but marketing also entails strategic decisions about the product itself, its price, and its placement. Taken together, these are often referred to as “the four Ps” [20]. While SSB producers are known to spend substantial amounts on marketing—Coca-Cola spent $3.96 billion US dollars on worldwide advertising alone in 2017 [21]—there is only fragmented peer-reviewed evidence describing the association between SSB marketing and consumption.

There is substantial evidence that marketing of food and drinks influences purchasing and consumption in children [22,23], who are less able to discern the persuasive intent of marketing [24]. However, little existing evidence focuses particularly on SSBs or adults, and most refers to a narrow range of drink products. There are also inherent methodological difficulties in ascribing a causal relationship between marketing and consumption; while observational, self-reported studies are at risk of reverse causation or confounding, sufficiently isolating the effect of marketing to conduct experimental studies, which is challenging and costly, and are often not reflective of real-world conditions in which marketing is consumed [25]. One way to increase confidence in a causal association is to demonstrate it in diverse international contexts. While SSB promotion utilizes increasingly diverse modalities, shifting away from traditional television advertisements towards digital media [26], there is less evidence available on the impact of non-television types of promotion of food and drink in general, and SSBs in particular [17]. Furthermore, despite international differences in SSB offerings, consumption, and regulation [27,28], we are not aware of any international comparisons of the relationship between promotion and consumption of SSBs. An absence of literature means it is currently unclear how well existing evidence concerning the relationship between SSB promotion and consumption generalizes to alternative countries and contexts.

With increasing interest in regulating the promotion of less healthy products [29], and concurrent awareness that other public health regulations, such as SSB taxes, might have unintended effects on SSB marketing [30], addressing gaps in the evidence base could inform future policy development. The study reported here aimed to address such gaps in existing literature by: (1) examining the association between total self-reported exposure to SSB promotion and SSB consumption in a large international sample of adults; (2) exploring whether this association differs by type of SSB promotion; and (3) investigating whether these relationships vary internationally between the UK, USA, Canada, Mexico, and Australia.

## 2. Materials and Methods

We conducted a cross-sectional analysis of data from an international survey to address our aims.

### 2.1. Study Design, Sampling, and Recruitment

Data were from the first wave of the International Food Policy Study (IFPS), a web-based survey completed in 2017 by adults aged 18–64 in the UK, USA, Canada, Mexico, and Australia. Most participants were recruited through the Nielsen Consumer Insights Global Panel and their partners’ panels. A random sample of panelists known to be eligible to take part in the study was sent email invitations. All Canadian participants aged 18–30 years, and some aged 31–32 years, were recruited separately from the parallel Canada Food Study (CFS), which was a preexisting online survey that formed the basis for the IFPS. Further details on the CFS are available elsewhere [31]. Prior to completing the survey, respondents provided consent, and their participation was incentivized using their panel’s existing reward structure. Further details of the study methodology can be found in the International Food Policy Study: Technical Report—Wave 1 (2017) at www.foodpolicystudy.com/methods [32]. Participants had to pass a data quality screening question to be included in the subsequent analysis.

### 2.2. Variables Used in the Analysis

#### 2.2.1. Sugar-Sweetened Beverage Consumption

The seven-day Beverage Frequency Questionnaire (BFQ) included in the study was used to derive an SSB consumption variable. The BFQ is a validated mode of dietary recall [33], which uses photographs to prompt respondents to recall the number and size of 22 types of drinks, including caloric and non-caloric, alcoholic and non-alcoholic beverages. First, participants were asked to report the number of drinks they had consumed within each beverage category in the past 7 days. Next, participants were shown an image with an array of container sizes with corresponding volumes (mL or fl oz) specific to each beverage category for which they had indicated any consumption. Container sizes varied for each country according to the products available in each market. If a respondent selected the lowest size category (“less than [smallest size]” option), this was recoded as half of the smallest listed option that had a specified size (e.g., 50% of 250 mL = 125 mL). Likewise, respondents reporting the highest size category of “more than [largest size]” were recoded as 125% of the largest option with a specified amount (e.g., 125% of 710 mL = 888 mL). Respondents who selected “Don’t know” or “Refused”, entered an implausibly large number of drinks consumed (>70), or failed to provide values for both the number and size for a particular drink were excluded from analyses. A volume variable was calculated for each beverage category by multiplying the derived drink size and frequency variables.

Total SSB consumption was computed by summing volumes for sugar sweetened drinks (see Table 1). The total SSB consumption variable was categorized into three groups: non-consumers, low consumers, and high consumers, based around the weighted median of weekly SSB consumption amongst consumers (survey population: 1830 mL; analytical sample: 1841 mL).

#### 2.2.2. Total Self-Reported Exposure to SSB Promotion

Participants were asked to report whether or not they had seen or heard 15 types of advertisements or promotions for sugary drinks in the last 30 days (including “other”; see Table 1), hereafter referred to as “promotions”. An aggregate variable of self-reported awareness of exposure to SSB promotion was computed by summing participants’ binary responses to each individual type of exposure, producing a variable with possible values between 0 and 15. Subsequent analyses treated this as a continuous variable.

#### 2.2.3. Self-Reported Exposure to Different Types of SSB Promotion

The aggregate self-reported promotion exposure variable was based on the assumption that exposure to different types of SSB promotions have homogenous associations with SSB consumption. To explore the potential for the association between SSB promotions and consumption to differ by type of promotion, the individual promotional exposures were also categorized into four dichotomous variables (see Table 1): exposure to traditional SSB promotions, exposure to digital SSB promotions, exposure to SSB promotions in the recreational environment, and exposure to SSB promotions in the functional environment. In this study, we defined “recreational environment” as the environment in which participants interact on the basis of enjoyment; whereas the “functional environment” is used by participants to complete a specific function or task. Self-reported exposure to “giveaways, samples, or special offers” was assigned to the recreational environment, though it could also be assigned to the functional environment; our findings were robust in reassigning this exposure between the two groups.

#### 2.2.4. Socio-Demographic Variables

Sociodemographic characteristics were self-reported in the survey. Since SSB consumption is known to vary across population sub-groups, those sociodemographic characteristics that have been reported elsewhere to be associated with SSB consumption were selected a priori to be included as covariates in the modelling. These were: country, age, and sex [34], ethnicity [35], and highest education level attained (as a proxy for socioeconomic status [35]).

#### 2.2.5. Ethics

The study received ethical approval from the University of Waterloo’s Research Ethics Committee (Office of Research Ethics# 21460 for the IFPS and Office of Research Ethics# 30893 for the CFS).

### 2.3. Analyses

All analyses were conducted using STATA 14.2. Survey participants providing valid responses to all of the variables used in the analyses were included in the analytical sample. Analyses were weighted with post-stratification sample weights rescaled to the analytical sample. For each country, these weights were constructed using population estimates from census data based on age, sex, and region. Applying sample weights throughout analyses helped to minimize the influence of differential non-response on the population representativeness of findings.

The sociodemographic characteristics of the sample were summarized. To test for differences between those included and excluded from the modelling, independent sample t-tests were used for continuous variables (total SSB promotion exposure, age) and Pearson’s χ^2^ tests for categorical variables (SSB consumption, types of SSB promotion, country, sex, ethnicity, education).

To examine the association between total self-reported exposure to SSB promotion and consumption, a multinomial logistic regression model was fit to the data using total exposure to SSB promotion as the independent variable and SSB consumption as the dependent variable, adjusting for country, sex, age, ethnicity, and education. Multinomial logistic regression modelling is appropriate in instances like these, where the dependent variable is nominal and has more than 2 categories, as illustrated elsewhere [36]. The UK was initially set as the reference country. To draw comparisons between included countries, the models were repeated by changing the reference country until all pairwise permutations were exhausted. To determine if the association between exposure to SSB promotion and SSB consumption varied by type of SSB promotion, we fitted a multinomial logistic regression model that included and mutually adjusted for all SSB promotion exposure groups (including exposure to “other” promotions, which was not analyzed as a separate dichotomous exposure), in addition to adjusting for the same covariates as described above. Finally, the first two models were extended to include multiplicative interactions between promotion exposures and country, in order to determine if associations between SSB promotion and consumption varied between countries. In the case of the second model, with the four different types of SSB promotion exposure, interactions with each type were introduced separately. The Wald test assessed the significance of these interaction terms; for countries with significant interaction terms, country-stratified models were produced.

## 3. Results

### 3.1. Sample Characteristics

Table 2 presents the sociodemographic characteristics of the analytical sample (after applying response weights). Of the 19,857 survey respondents, 78% (15,515) were included in the analyses. A total of 4342 individuals were excluded due to inadequate data: They refused to answer (or answered “don’t know”), had discrepancies in their responses, or had missing data for at least one of the variables in the models. Within the analytical sample, 51% (7862) were men, the median age was 40 years, 79% (12,248) identified with majority ethnic groups, and approximately half had high education attainment (55%; 8516). After finding the weighted median SSB intake volume, consumption was approximately evenly distributed across the three groups in the analytical sample: 5265 (34%) were non-consumers, 5117 (33%) were low consumers, and 5133 (33%) were high consumers. Overall, 29% (4549) of the study population self-reported no exposure to SSB promotions. No respondents reported exposure to all 15 types of promotion. Among those reporting exposure to SSB promotions, there was a median value of 3 exposures; 9322 (60%) reported exposure to traditional promotions, 5565 (36%) to digital promotions, 4363 (28%) to promotions in the recreational environment, and 5265 (34%) to promotions in the functional environment.

Differences were found between study participants included and excluded from the analytical sample (see Supplementary Table S1). For example, individuals in the analytical sample had lower total exposure to SSB promotions than those excluded and were older. We chose not to impute missing values because the magnitude of these differences was small.

### 3.2. Total Exposure to SSB Promotions and SSB Consumption

**Figure 1:**
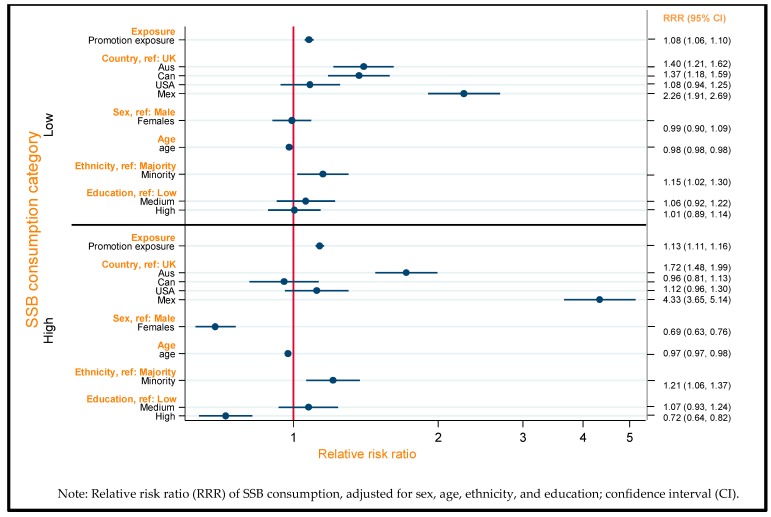
Forest plot summarizing associations between self-reported exposure to SSB promotions and SSB consumption, estimated by multinomial logistic regression with adjustment for sociodemographic characteristics (*n* = 15,515; ref: no SSB consumption).

Figure 1 summarizes the associations between total exposure to SSB promotions and SSB consumption after adjustment for socio-demographic characteristics. The likelihood of being a low or a high SSB consumer (relative to a non-consumer) increased as self-reported exposure to promotions increased (low: relative risk ratio (RRR) = 1.08, 95% confidence interval (CI) = 1.06–1.10; high: RRR = 1.13, 95% CI = 1.11–1.16), compared with being a non-consumer. There were also associations between some of the sociodemographic characteristics included as covariates and SSB consumption. Women (compared to men) and people with high educational attainment (compared to low attainment) were less likely to be high SSB consumers. Meanwhile, younger individuals and ethnic minorities (compared to majorities) were more likely to be low or high SSB consumers.

Country variations in consumption were also present and are described in Table 3. Compared with the UK, Australian (RRR = 1.40, 95% CI: 1.21–1.62), Canadian (RRR = 1.37, 95% CI: 1.18–1.59), and Mexican (RRR = 2.26, 95% CI: 1.91–2.69) participants were more likely to be low SSB consumers than non-consumers. This pattern persisted for likelihood of high SSB consumption for Australia (RRR = 1.72, 95% CI: 1.48–1.99) and Mexico (RRR = 4.33, 95% CI: 3.65–5.14) compared to no consumption. USA participants were less likely than Australian (RRR = 0.77, 95% CI: 0.67–0.89) and Canadian participants (RRR = 0.77, 95% CI: 0.68, 0.87) to be low SSB consumers than non-consumers, but this only persisted at high SSB consumption for Australia (RRR = 0.65, 95% CI: 0.56–0.75). Mexican participants were more likely to be low or high consumers than non-consumers compared with all countries included in the study.

### 3.3. Exposure to Different Types of SSB Promotion and SSB Consumption

**Figure 2:**
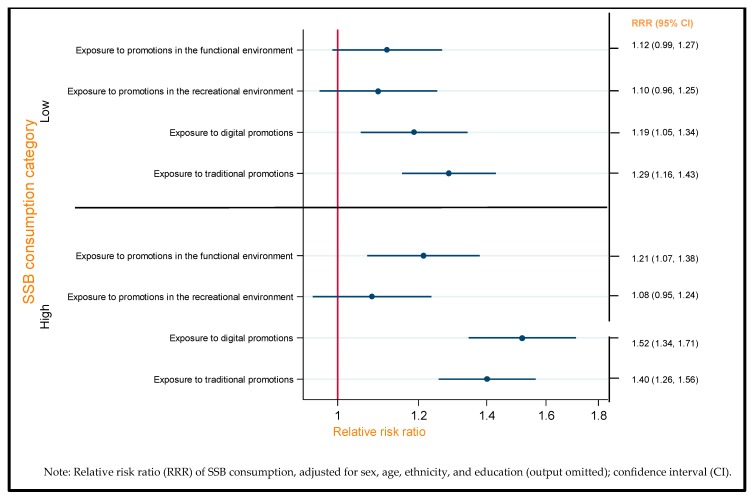
Forest plot summarizing the association between self-reported exposure to different types of SSB promotions and SSB consumption, estimated by multinomial logistic regression with adjustment for sociodemographic characteristics (*n* = 15,515; ref: no SSB consumption).

Figure 2 shows the associations between exposure to different types of SSB promotion and SSB consumption, after adjustment for socio-demographic factors. Increased exposure to digital promotions was associated with increased likelihood of both low and high SSB consumption compared with non-consumption (low: RRR = 1.19, 95% CI: 1.05–1.34; high: RRR = 1.52, 95% CI: 1.34–1.71), and similarly for traditional promotions (low: RRR = 1.29, 95% CI: 1.16–1.43; high: RRR = 1.40, 95% CI: 1.26–1.56). Exposure to SSB promotion in the functional environment was only significantly associated with a likelihood of high SSB consumption (RRR = 1.21, 95% CI: 1.07–1.38), compared with no SSB consumption.

### 3.4. Country Variations in the Relationships between Exposure to SSB Promotion and SSB Consumption

There was inconsistent evidence to show that associations between exposure to SSB promotions and SSB consumption varied across countries. There was no evidence that the relationship between total exposure to SSB promotions and SSB consumption (*p* = 0.36) varied between countries, nor for the relationship between exposure to promotions in the functional environment and SSB consumption (*p* = 0.07). However, there was some evidence that the relationships between exposure to digital promotions, traditional promotions, and promotions in the recreational environment and SSB consumption varied between countries (*p* < 0.0001 respectively). To elucidate these differences, we fitted country-stratified models, which are summarized in Supplementary Table S2. In short, only the USA and Mexico had significant associations with both low and high SSB consumption when exposed to digital promotions; the UK was significant at high SSB consumption only. For exposure to traditional promotions, only the USA and Australia had significant associations with both low and high SSB consumption; Canada was significant at only low SSB consumption. For exposure to promotions in the recreational environment, there were no significant country-stratified associations with SSB consumption.

## 4. Discussion

### 4.1. Summary of Findings

To our knowledge, this is the first study to explore the relationship between self-reported exposure to a range of SSB promotions and SSB consumption in a diverse international sample of adults and whether this differs by type of exposure or country. We found that increased self-reported exposure to SSB promotions was associated with a greater likelihood of SSB consumption. Of the various types of promotions investigated in the study, exposure to digital and traditional promotions had the strongest associations with SSB consumption. We also found some evidence that the strength of association between self-reported exposure to digital promotions, traditional promotions, and promotions in the recreational environment and SSB consumption varied between countries in the study.

### 4.2. Strengths and Limitations of Methods

The strengths of the IFPS design have been reported elsewhere [37]. Using IFPS data facilitated the inclusion of a large and diverse population sample of adults from countries with varying efforts to reduce exposure to beverage marketing and reduce consumption of sugary drinks. This increases the generalizability of our findings. Unlike existing literature that tends to focus on specific forms of promotion (for example, television advertising [27]), we included a broad range of promotion exposures. Using the validated BFQ [33] increased the internal validity of our results. However, recruitment using non-probability sampling limited the ability of our analyses to provide nationally representative findings, as did sociodemographic differences between participants in the total and analytical sample. Applying sampling weights throughout helped reduce the threat to generalizability; imputation may have further reduced the potential for this to introduce bias. All variables were self-reported, which could introduce recall and social desirability bias [38]. The potential for social desirability bias was minimized by collecting data online [39]. The survey was conducted in December; early winter in the UK, USA, Canada, and Mexico, but early summer in Australia. As SSB intake is likely to be higher in summer [40], this may introduce differential seasonal effects between countries. It is also unclear how participants interpreted the questions on exposure to SSB ‘advertising and promotions’; some may have employed more expansive definitions to include marketing via product, price, and placement. This may have introduced further bias if there were systematic differences in how people interpreted the questions, according to either their exposure or SSB consumption. Finally, measures of exposure to promotions were not validated, albeit similar questions have been used elsewhere [41].

### 4.3. Comparison to Previous Research and Interpretation of Findings

Our findings extend existing studies of exposure to SSB promotions and consumption, which have tended to focus on specific sociodemographic groups, for example, young people and children [42,43,44]. Our findings that self-reported exposure was positively associated with SSB consumption is consistent with the limited existing epidemiological evidence that focuses specifically on this topic [45,46,47,48,49], as well as the wider literature, which reports a more generic association between exposure to food and drink marketing and consumption in general [23]. The results extend previous work on exposure to SSB promotion by including participants across sociodemographic groups and including countries with different food policies. That the overall finding of an association between self-reported marketing exposure and SSB consumption did not vary between countries with different food environments and policy contexts increases confidence that this is a generalizable finding. Our cross-sectional analyses were unable to demonstrate any putative causal pathways, and it is possible that reverse causation is operating with higher SSB consumers being more likely to notice and so report marketing exposure. However, the high expenditure of soft-drinks firms on marketing provides support for the hypothesis that exposure to promotions of SSBs leads to their consumption. The interpretation of a causal relationship is further supported by numerous experimental studies on exposure to both SSB marketing specifically and food and drink marketing more generally [23].

The fact that the study’s findings depend on self-reported exposure to SSB promotions adds another dimension to their interpretation. Assessing exposure using a self-report method has been used in similar research examining the relationship between components of marketing and consumption [41]. However, memory of marketing exposure is multidimensional, of which recall and recognition might only capture a small proportion [50]; specifically, the extent that advertising messages have been encoded in memory and the ability to access that information [51]. Meanwhile, consumers are typically less good at gauging the influence of marketing on implicit attitudes [52], even though some evidence suggests that implicit memory may be more important than explicit recall to soft drink choice [53]. In this light, it is unclear how well our results reflect the association between implicit influences of sugary drinks promotion and marketing more generally and SSB consumption. Future research could address this by seeking to comprehensively measure exposure to all components of marketing; albeit these methods might come with their own logistical challenges and a risk of overestimating implicit exposure.

To our knowledge, evidence of variation in the strength of association between exposure to SSB promotions and consumption is a novel finding. We found that self-reported exposure to more interactive modes of promotion (digital and traditional) were associated with greater SSB consumption. In contrast, exposure to promotion in the environment that may be more passively consumed were it not conclusively associated with SSB consumption [54]. This may relate to the volume or intensity of exposure that participants experienced within each of these modes. Whilst we measured any exposure versus none, we did not capture variations in volume and intensity, and these may have been greater amongst those reporting exposure to any digital and traditional promotions. We also did not associate our findings with the fact that most promotion expenditure tends to go towards traditional promotion (e.g., television [55]), and increasingly towards digital [26]. Interactive modes of promotion may also be more likely to be recalled than others [51]. Measuring and quantifying marketing exposure using more objective methods, as attempted elsewhere [56], may help to distinguish between these possibilities.

Our attempts to differentiate exposure to different types of promotion may also be overly simplistic. Firms are concerned with building brand “equity through marketing: “everything that exists in the minds of the customer with respect to a brand (e.g., thoughts, feelings, experiences, images, perceptions, beliefs, and attitudes)” [57]. This means that firms rely on different components of marketing to work together synergistically, rather than assuming different and separate effects of different modalities. Future research could seek to use brand-level data to understand how different forms of SSB marketing—including promotion—work synergistically to influence SSB consumption.

Existing evidence from the IFPS shows that exposure to SSB promotions varies by country [28], but clarifying whether the association between marketing exposure and SSB consumption differs by country is important for translating the understanding of SSB consumption and regulation internationally. There are several possible explanations for inconsistent evidence of country variations in this study, including the fact that the content of these SSB promotions are likely to vary by country. Given that the effectiveness of marketing is understood to be a function of exposure and persuasive power [58], cross-country differences in the volume of exposure or persuasive power for these promotions may account for the small variations we found in this dataset. Repeating these analyses with a more diverse set of countries, incorporating a measure for persuasive power (e.g., persuasive techniques [59]), and attempting to understand the extent to which this varies by the company enacting the marketing, could further clarify these variations. This could inform public health policymakers wishing to know whether they should apply international evidence to inform local policy decisions.

In addition to answering the central research questions, analyses also provided insight into the relationship between sociodemographic characteristics and SSB consumption. SSB consumption was higher among males, people with lower educational attainment, younger individuals, and ethnic minorities. All these findings are consistent with wider literature [35],increasing our confidence in the external validity of our main findings.

### 4.4. Implications of Findings and Future Research

Substantial existing evidence links SSB consumption with poor health outcomes [14,15]. By finding an association between exposure to SSB promotions and SSB consumption, this study adds weight to existing calls to restrict SSB marketing for the benefit of public health [26]. To provide further justification for such restrictions, future research should clarify whether this relationship is causal and extends to other components of marketing. As “mere exposure theory” suggests that product choice may be mediated by repetition of exposure to marketing [53], future studies could extend the present research to quantify and understand the effects of the degree of exposure to promotion. The wear-out effects of repetition of marketing exposure could also be studied by exploring non-linear relationships between marketing exposure and consumption [53], which was not accounted for in the present study. Further developing understanding of the relationship between SSB marketing and consumption could help policymakers develop multicomponent strategies to address SSB consumption, such as that currently being modelled in Chile [60]. For example, if SSB taxes lead to unintended changes to marketing, they could be introduced alongside greater SSB marketing restrictions.

Our results also provide some evidence that SSB marketing restrictions should particularly focus on traditional and digital forms of promotion. Further research should seek to confirm this and explore causal mechanisms for the variations in the effects we found between different types of promotion exposure. The possibility of different types of marketing working synergistically to affect consumption should also be explored. However, our findings suggest that current attempts to restrict SSB marketing that focus overwhelmingly on TV advertising [61] could be undermined by reactive increases in say, digital advertising, which are similarly associated with SSB consumption. Taking a broader approach to marketing regulation might ultimately prove more effective.

Given that competitive edge depends on novelty, we should expect to see continued innovation in ways that sugary drinks firms seek to promote their products (such as advergaming [17]), and future research should seek to understand the impact of this on SSB consumption. While we found inconsistent evidence of the difference in the strength of association between self-reported exposure to SSB promotion and consumption between the countries included, future research could test this in a more heterogeneous set of countries.

## 5. Conclusions

We found a positive association between self-reported exposure to SSB promotions and SSB consumption among adults in the UK, Mexico, Australia, Canada, and the USA. The strength of this association varied by type of promotion exposure: Exposure to digital and traditional promotions was associated with greater SSB consumption, whilst there was a mixed picture of associations for promotions in the functional and recreational environment. There was also some evidence that these associations varied by country. Using a self-reported measure of exposure and not measuring the intensity of exposure are key limitations of the study. Nonetheless, efforts to restrict marketing of SSBs should focus on a wider range of marketing than just TV promotions and should reflect country context.

## Figures and Tables

**Table 1:** Descriptions of the variables and question wording from the International Food Policy Study (IFPS) (2017).

Variable	Question	Relevant Response Options	Used in Analysis
SSB consumption	[Calculated from the Beverage Frequency Questionnaire: Reported frequency and volume of consumption over the last 7 days]	Regular pop; sweetened fruit drinks; regular flavored water with calories; regular sports drinks; regular energy drinks; chocolate milk or other flavored milk; specialty coffees; sweetened smoothies, protein shakes, or drinkable yogurts	Non-consumers (of these options); low SSB consumers (<overall median); high SSB consumers (>overall median)
Total self-reported exposure to SSB promotion	Sugary drinks are drinks that contain added sugar, such as fizzy drinks (Australia: soft drinks; Canada: pop), fruit drinks, sports drinks, energy drinks, chocolate milk, and speciality coffees that have added sugar. In the past 30 days, have you seen or heard any advertisements or promotions for SUGARY DRINKS in the following places? (select all that apply)	TV ads; radio ads; online/internet ads; mobile app/video game; social media (e.g., Twitter, Facebook, Snapchat); in a text message; magazine or newspaper; billboard or outdoor sign (e.g., posters, transit ads); in movies; at school/on campus; signs or displays in stores or restaurants; at a recreation/community center; sports event or sponsorship (e.g., logos or links with events, teams, or athletes); giveaways, samples, or special offers; other; I haven’t seen any marketing for sugary drinks in the last 30 days; don’t know; refuse to answer	Summed number of locations responded positively to, with those responding: “I haven’t seen any marketing for sugary drinks in the last 30 days”, coded to 0, and “Don’t know” and “refuse to answer” set to missing
Exposure to traditional SSB promotion	[Same wording as that for “Total self-reported exposure to SSB promotion” variable]	TV ads; radio ads; in a text message; magazine or newspaper	“Yes” if self-reported exposure to advertisements or promotions for SUGARY DRINKS in any of these locations, “No” if not
Exposure to digital SSB promotion	[Same wording as that for “Total self-reported exposure to SSB promotion” variable]	Online/internet ads; mobile app/video game; social media (e.g., Twitter, Facebook, Snapchat)	“Yes” if self-reported exposure to advertisements or promotions for SUGARY DRINKS in any of these locations, “No” if not
Exposure to recreational environment SSB promotion	[Same wording as that for “Total self-reported exposure to SSB promotion” variable]	Films or cinema; giveaways, samples, or special offers; at a recreation/community center; at a sports event or concert	“Yes” if self-reported exposure to advertisements or promotions for SUGARY DRINKS in any of these locations, “No” if not
Exposure to functional environment SSB promotion	[Same wording as that for “Total self-reported exposure to SSB promotion” variable]	Billboard or outdoor sign; at a school/college/university; signs or displays in supermarkets, convenience shops or restaurants	“Yes” if self-reported exposure to advertisements or promotions for SUGARY DRINKS in any of these locations, “No” if not
Country	Automatically assigned	UK; Canada; Australia; USA; Mexico	UK; Canada; Australia; USA; Mexico
Sex	What sex were you assigned at birth, meaning on your original birth certificate?	Male; female	Male; female
Age	How old are you?	In years	Continuous
Ethnicity	Which of the following best describes your ethnic or racial background?	[Ethnicity options particular to each country]	Majority group; minority group
Education	What is the highest level of education you have completed?	Below upper secondary schooling = low; upper secondary schooling = medium; tertiary = high	Low; medium; high

**Table 2:** Characteristics of the IFPS (2017) analytical sample (*n* = 15,515), post-weighting.

Variable	Level	*n*	%
**SSB consumption**	None	5265	34
	Low	5117	33
	High	5133	33
**Total exposure to SSB promotion**	Continuous	(none = 4549)	Median = 3 (IQ 1, 5)
**Exposure to traditional SSB promotion**	Yes	9322	60
	No	6193	40
**Exposure to digital SSB promotion**	Yes	5565	36
	No	9950	64
**Exposure to recreational environment SSB promotion**	Yes	4363	28
	No	11152	72
**Exposure to functional environment SSB promotion**	Yes	5265	34
	No	10,250	66
**Country**	UK	3026	20
	Australia	2996	19
	Canada	2575	17
	USA	3793	24
	Mexico	3126	20
**Sex**	Male	7862	51
	Female	7653	49
**Age (years)**	Continuous	15,515	Median = 40 (IQ 29, 52))
**Ethnicity**	Majority	12,248	79
	Minority	3267	21
**Education attainment**	Low	3108	20
	Medium	3891	25
	High	8516	55

Note: For continuous variables, *n* refers to the total number of participants who had a value of the variable and the median and interquartile ranges (IQ) are presented instead of %.

**Table 3:** Between country contrasts of the associations between total self-reported exposure to SSB promotions and SSB consumption using multinomial logistic regression (*n* = 15,515, ref: no SSB consumption, only country estimates printed).

	Likelihood of SSB Consumption Compared with No Consumption		
	RRR	Low 95% CI	High 95% CI
No SSB Consumption (*n* = 5265), ref			
Low SSB Consumption (*n* = 5117)			
Australia vs. UK	1.40	1.21	1.62
Canada vs. UK	1.37	1.18	1.59
USA vs. UK	1.08	0.94	1.25
Mexico vs. UK	2.26	1.91	2.69
Canada vs. Australia	0.98	0.84	1.13
USA vs. Australia	0.77	0.67	0.89
Mexico vs. Australia	1.62	1.36	1.93
USA vs. Canada	0.77	0.68	0.87
Mexico vs. Canada	1.68	1.45	1.95
Mexico vs. USA	2.09	1.77	2.47
High SSB consumption (*n* = 5133)			
Australia vs. UK	1.72	1.48	1.99
Canada vs. UK	0.96	0.81	1.13
USA vs. UK	1.12	0.96	1.30
Mexico vs. UK	4.33	3.65	5.14
Canada vs. Australia	0.56	0.47	0.65
USA vs. Australia	0.65	0.56	0.75
Mexico vs. Australia	2.52	2.13	2.99
USA vs. Canada	1.08	0.95	1.24
Mexico vs. Canada	4.40	3.78	5.13
Mexico vs. USA	3.88	3.30	4.56

Note: Relative risk ratio (RRR), confidence interval (CI), adjusted for sex, age, ethnicity, and education. Reference country presented second.

## Supplementary Materials

The following are available online at https://www.mdpi.com/2072-6643/11/12/3047/s1, Table S1: Differences between individuals included and excluded from the analytical sample, pre-weighting, Table S2: Country-stratified models of the association between total exposure to SSB promotions and SSB consumption using multinomial logistic regression (*n* = 15,515; ref: no SSB consumption, only country estimates printed).
