# Supplementary material for: The Relationship between Self-Reported Exposure to Sugar-Sweetened Beverage Promotions and Intake: Cross-Sectional Analysis of the 2017 International Food Policy Study
Source: Nutrients. 2019 Dec 13;11(12):3047. doi: 10.3390/nu11123047 (PMC6950183; doi:10.3390/nu11123047)
Supplement: Supplementary file 1 [file nutrients-11-03047-s001.zip › Supplementary Table S1.pdf]

Supplementary Table S1: Differences between individuals included and excluded from the analytical sample, pre-weighting

Variable	Analytical sample, n (%)	Excluded, n (% [c])	χ^2 or t-value [b]	p-value
**SSB consumption [a]**	15515	4342	17.86	<0.001
None	5149 (33)	421 (39)	-	-
Low	5182 (33)	348 (32)	-	-
High	5184 (33)	308 (29)	-	-
Missing	-	3265	-	-
**Total SSB promotion exposure [b]**	15515	4342	-3.86	0.0001
Missing	-	1139	-	-
**Exposure to promotion in the functional environment [a]**	15515	4342	0.09	0.768
Yes	5331 (34)	1098 (34)	-	-
No	10184 (66)	2123 (66)	-	-
Missing	-	1121	-	-
**Exposure to promotion in the recreational environment [a]**	15515	4342	0.09	0.760
Yes	4443 (29)	931 (29)	-	-
No	11072 (71)	2290 (71)	-	-
Missing	-	1121	-	-
**Exposure to digital promotions [a]**	15515	4342	35.57	<0.001
Yes	5847 (38)	1395 (43)	-	-
No	9668 (62)	1826 (57)	-	-
Missing	-	1121	-	-
**Exposure to traditional promotions [a]**	15515	4342	23.43	<0.001
Yes	9437 (61)	2106 (65)	-	-
No	6078 (39)	1115 (35)	-	-

Missing	-	1121	-	-
**Country** [a]	15515	4342	86.77	<0.001
UK	2999 (19)	1048 (24)	-	-
Australia	3021 (19)	746 (17)	-	-
Canada	2570 (17)	548 (13)	-	-
USA	3815 (25)	1053 (24)	-	-
Mexico	3110 (20)	947 (22)	-	-
**Sex** [a]	15515	4342	42.12	<0.001
Males	7340 (47)	1813 (42)	-	-
Females	8175 (53)	2529 (58)	-	-
**Age** [b]	15515	4342	8.22	<0.001
**Ethnicity** [a]	15515	4342	8.36	0.004
Majority	12412 (80)	3200 (78)	-	-
Minority	3103 (20)	905 (22)	-	-
Missing	-	237	-	-
**Education** [a]	15515	4342	115.64	<0.001
Low	3068 (20)	1106 (26)	-	-
Medium	3826 (25)	1106 (26)	-	-
High	8621 (56)	1976 (47)	-	-
Missing	-	154	-	-

Notes
19,857 individuals in the survey population. Individuals included in the analytical sample: n=15,515; excluded: n=4,342.Weights were not applied to these analysis.

[a] Pearson's χ^2 tested differences between those included and excluded from the analytical sample; [b] Independent samples t-test tested differences, t value for mean included– mean excluded from sample; [c] percentages are taken with missing values excluded from the total.
