# Supplementary material for: The Relationship between Self-Reported Exposure to Sugar-Sweetened Beverage Promotions and Intake: Cross-Sectional Analysis of the 2017 International Food Policy Study
Source: Nutrients. 2019 Dec 13;11(12):3047. doi: 10.3390/nu11123047 (PMC6950183; doi:10.3390/nu11123047)
Supplement: Supplementary file 1 [file nutrients-11-03047-s001.zip › Supplementary Table S2.pdf]

Supplementary Table S2: Country-stratified models of the association between total exposure to SSB promotions and SSB consumption using multinomial logistic regression (n=15,515; ref: no SSB consumption, only country estimates printed)

Type of promotion exposure	Country	Likelihood of SSB consumption compared with no consumption		
		RRR	Low 95% CI	High 95% CI
**Exposure to SSB promotions in the recreational environment**				
No SSB consumption (n=5,265), ref				
Low SSB consumption (n=5,128)	UK	1.27	0.88	1.82
	Australia	1.06	0.77	1.46
	Canada	1.11	0.83	1.47
	USA	1.22	0.95	1.57
	Mexico	0.88	0.64	1.23
High SSB consumption (n=5,123)	UK	1.30	0.91	1.86
	Australia	1.07	0.78	1.47
	Canada	1.30	0.95	1.77
	USA	1.08	0.83	1.40
	Mexico	0.92	0.67	1.26
**Exposure to digital SSB promotion**				
No SSB consumption (n=5,265), ref				
Low SSB consumption (n=5,128)	UK	1.13	0.84	1.51
	Australia	0.92	0.69	1.24
	Canada	1.02	0.78	1.34
	USA	1.38	1.09	1.74
	Mexico	1.38	1.04	1.85
High SSB consumption (n=5,123)	UK	1.99	1.49	2.66
	Australia	1.20	0.91	1.59
	Canada	1.18	0.88	1.59
	USA	1.74	1.37	2.22
	Mexico	1.60	1.22	2.09
**Exposure to traditional SSB promotion**				
No SSB consumption (n=5,265), ref				
Low SSB consumption (n=5,128)	UK	1.12	0.89	1.40
	Australia	1.46	1.17	1.83
	Canada	1.28	1.00	1.63
	USA	1.35	1.09	1.69
	Mexico	1.13	0.80	1.59
High SSB consumption (n=5,123)	UK	1.22	0.97	1.54
	Australia	1.39	1.11	1.73
	Canada	1.25	0.95	1.64
	USA	1.64	1.30	2.07
	Mexico	1.32	0.96	1.83

Note: RRR, relative risk ratio, adjusted for all types of marketing, sex, age, ethnicity and education (omitted).
